# Three-dimensional printed cast assisted screw fixation of calcaneal fractures: a prospective study

**DOI:** 10.1186/s12891-023-06927-4

**Published:** 2023-10-10

**Authors:** Qizhi Song, Tao Li, Huan Xia, Yan Li, Chengbin Feng, Yajun Lin, Huahong Wang, Jinbiao Hu, Qilong Jiang

**Affiliations:** 1 Department of Orthopaedic Surgery, Chonggang General Hospital, Chongqing, China; 2 Nursing Department, Chonggang General Hospital, Chongqing, China; 3 Central Sterile Supply Department, Chonggang General Hospital, Chongqing, China; 4 Department of Orthopaedic Surgery, Chongqing Orthopedic Hospital of Traditional Chinese Medicine, No. 9, Jiefang West Road, 400010 Chongqing, China

**Keywords:** Calcaneal fractures, Less invasive, Screw fixation, 3D printing

## Abstract

**Background:**

Treatment of displaced intra-articular calcaneal fractures (DIACFs) with percutaneous screw fixation remains defective in some aspects. A novel three-dimensional (3D) printed cast was devised to assist screw placement. This study assessed the radiological and functional outcomes of 3D-printed cast assisted screw fixation for patients with DIACFs.

**Methods:**

Patients with unilateral Sanders type II or III DIACFs admitted to a single-centre hospital underwent either 3D-printed cast assisted screw fixation (3D group) or minimally invasive plate fixation (control group) from September 2020 to November 2022. All patients were assessed at one, two, three, and six months of follow-up. Comparison between groups was conducted in operative duration, fluoroscopic times, radiographic measurements of the calcaneus, and the American Orthopaedic Foot and Ankle Society (AOFAS) Ankle-Hindfoot Score.

**Results:**

A total of 32 patients were enrolled (19 in the 3D group versus 13 in the control group). Significant differences were detected between the 3D group and control group in operative duration (53.63±8.95 min, 95.08±8.31 min, *P* <0.001), fluoroscopic times (7.37±1.21, 16.85±1.57, *P* <0.001). At a follow-up of six months, the 3D group showed better restoration than the control group in calcaneal width, height, Bohler angle, and AOFAS Ankle-Hindfoot scores (all *P* <0.001). No significant differences were shown in calcaneal length and Gissane angle (*P* >0.05). No wound-related complications occurred in either group.

**Conclusion:**

The 3D-printed cast assisted screw fixation has shown superiority over minimally invasive plate fixation in the operative duration, fluoroscopic exposure, morphological restoration of the calcaneus, and functional outcomes in the treatment of DIACFs.

## Introduction

Calcaneal fractures most frequently occur in tarsal injury, among which approximately 75% of the subtalar facets are affected [1, 2]. The optimal treatment of displaced intra-articular calcaneal fractures (DIACFs) remains controversial. Surgical modality with open reduction and plate fixation using an extensile lateral approach has been prevailing for decades [3]. However, the occurrence of postoperative wound complications accounts for as high as 30% of operated calcaneus using this approach [4]. With the application of a minimally invasive sinus tarsi approach, the prevalence of wound infection and necrosis has decreased, which was reported to range from 7.5 to 13.6% in several studies [5–8]. The management of wound healing poses a challenge to the orthopaedic surgeon. The relevant irritation of screws and plate could further compromise the ability to return to work. More recently, percutaneous reduction and screw fixation have been advocated for DIACFs, with the advantages of higher clinical satisfaction and lower complication rate [9–11]. Compared to traditional plate fixation, percutaneous screw fixation still exhibits several flaws including inadequate stability, loss of reduction, non-generally accepted screw configurations, tedious adjustment of screw insertion, and excessive fluoroscopic exposure [12, 13]. With the advent of three-dimensional (3D) printing technology, preoperatively printed templates have been widely applied to formulate surgical strategies of screw configurations [9, 14, 15]. Yet there is no report regarding intraoperative usage of the 3D-printed jig in assisting screw insertion. Given that the treatment of DIACFs with percutaneous screw fixation remains defective in some aspects, we devised a novel 3D-printed cast to assist screw insertion for calcaneal fractures. The current research aimed to evaluate radiological and functional outcomes of 3D-printed cast assisted screw fixation for patients with DIACFs. We hypothesized that the 3D printing-assisted surgical protocol would yield superior outcomes over minimally invasive plate fixation in treating DIACFs.

## Methods

The Institutional Ethics Committee of the Chonggang General Hospital (Chongqing, China) approved the current research (2020-KY-02). Informed consent from all involved patients has been obtained.

### Participants

From September 2020 to November 2022, all patients with calcaneal fractures admitted to our hospital were checked for eligibility. Inclusion criteria were patients (1) with unilateral Sanders type II or III fractures via computed tomography (CT) assessment, (2) aged more than 18 years. Exclusion criteria included (1) pathological fractures, (2) open calcaneal fractures, (3) concomitant trauma, (4) comminuted or displaced sustentaculum tali fractures, (5) fractured calcaneus with surgical history or prior deformity, (6) severe osteoporosis (T value < -3.0). Finally, a total of 32 patients (32 calcanea) were enrolled and assigned at the surgeon’s discretion to undergo the 3D-printed cast assisted percutaneous screw fixation (3D group) or plate fixation via a sinus tarsi approach (control group). The demographics are presented in Table 1.

**Table 1:** Demographic data of patients

	3D group (n = 19)	Control group (n = 13)	Statistics	*P* value
Gender			χ^2^ = 1.000	0.607
Male	12	8		
Female	7	5		
Age (years)	41.3±11.7	38.2±12.5	*t* = 0.700	0.489
Side of injury				
Left	11	7	χ^2^ = 0.051	0.821
Right	8	6		
Sanders classification			χ^2^ = 1.166	0.280
Type II	11	5		
Type III	8	8		
Type of injury			χ^2^ = 0.729	0.694
falling	12	9		
traffic injury	6	4		
other	1	0		

### Manufacture of 3D-printed cast

**Fig. 1:**
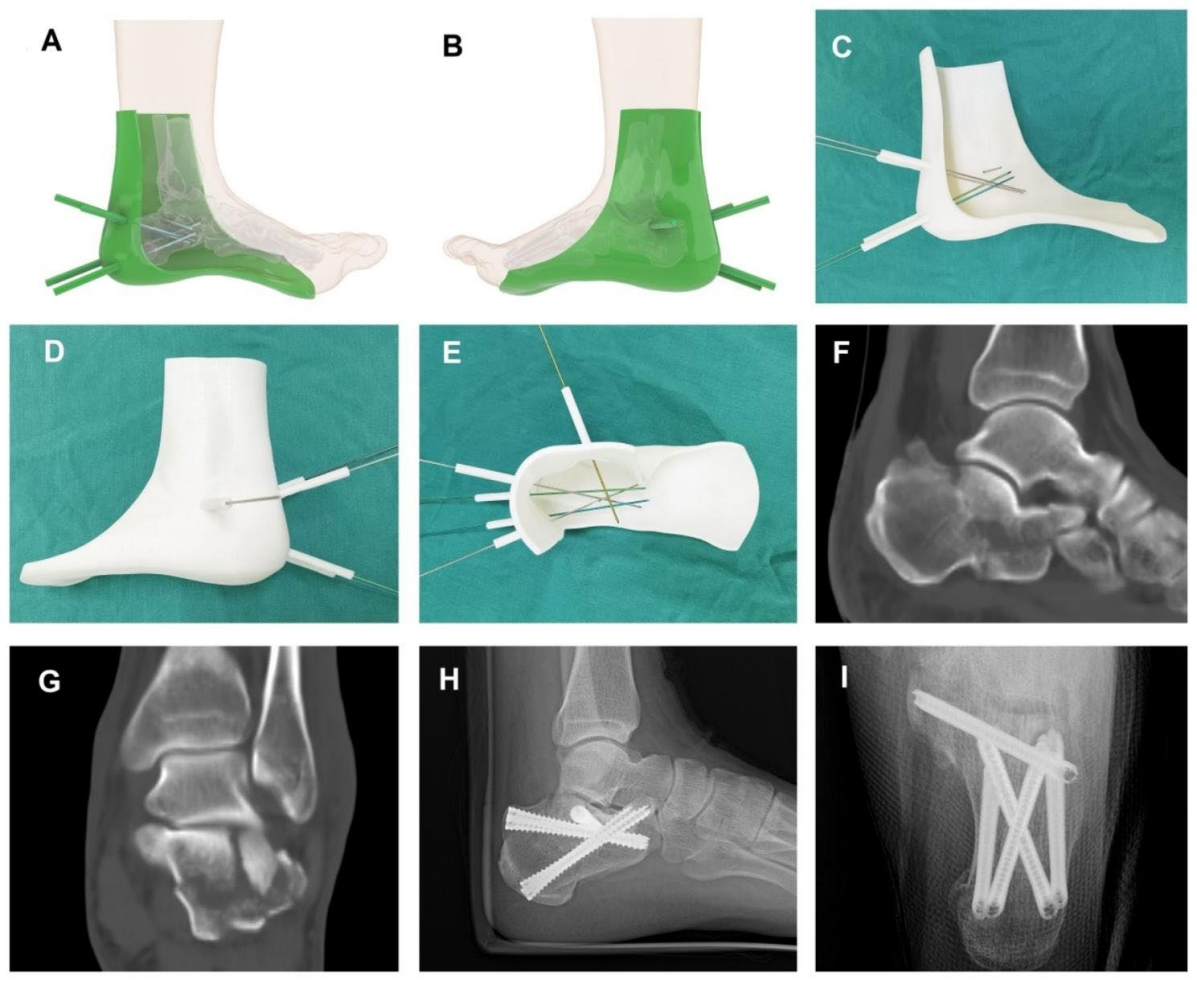
The design, fabrication, and application of 3D printed cast. **A,B** The reconstruction and design of 3D model based on CT data. **C, D, E** The 3D printed cast with five cannulas for insertion of 1.5 mm K-wires. **F, G** CT images of a patient (male, 42 years of age) with displaced intra-articular calcaneal fracture, showing sustentaculum tali nondisplaced. **H, I** A fractured calcaneus treated with percutaneous reduction and 3D printed cast assisted screw fixation

The 3D-printed cast is designed to assist percutaneous cannulated screw insertion. The affected ankle and foot were scanned within two days prior to surgery. The raw data were stored in DICOM format. The reconstruction and design of the 3D model were performed with the application of Mimics Research 19.0 (Materialise, Belgium), Geomagic Studio (Raindrop, USA), and Rhino 6 (Robert McNeel, USA). The extent of 3D coverage ranged from the distal plantar to the lateral malleolus. Five cannulas were attached to the 3D jig to guide the insertion of 1.5 mm K-wires. Based on the screw configuration, these five cannulas were classified into three groups. (1) A lateral cannula for sustentaculum tali fixation (Transverse screw). The insertion originated laterally 5 mm below the posterior facet and ended at the medial subcortical bone of sustentaculum tali. Entrance into the subtalar joint space should be avoided. (2) Two X-shaped trajectories (Crossed screws) ran obliquely from the superior calcaneal tuberosity to the inferior calcaneocuboid joint. (3) Two parallel trajectories (Parallel screws) ran obliquely from the inferior calcaneal tuberosity to the superior calcaneocuboid joint. The nondisplaced sustentaculum tali and calcaneocuboid joint were referenced for design. Then, the definitive 3D jig was printed within 48 h prior to surgery via the Creality Slicer-FDM 3D printer (Creality, China), with the use of polylactic acid (PLA) printing material. Finally, a sterilized 3D jig was prepared before surgery. The manufacture of the 3D jig is shown in Fig. 1.

### Interventions

For patients in the 3D group, the tissue swelling of the affected foot was not a contraindication of the operation. Following epidural anaesthesia, patients were turned into the lateral decubitus position. The operated foot should be placed upwards to carry out intraoperative fluoroscopy handily. Several Schanz pins were inserted into calcaneal fragments to perform the closed reduction. Under fluoroscopic guidance, the calcaneal length, width, height, angle, and articular congruity should be restored. If closed reduction failed, a minimally invasive sinus tarsi approach was adopted to return the subtalar facet under direct visualization. After an achievement of satisfactory reduction, the calcaneal fragments were fixed temporarily by K-wires. Then, the 3D-printed cast was attached to the affected foot. Five 1.5 mm K-wires passed through the predesigned cannulas respectively. The tip of the K-wire should be limited beneath the subcortical bone of the calcaneus. C-arm assisted fluoroscopic imaging was used to ensure satisfactory K-wire configuration. It is possible to make minor adjustments until optimal insertion is reached. Subsequently, five fully threaded cannulated screws (Wego, China) were placed along the K-wires respectively. Lateral and axial heel views were taken to determine the eventual fixation quality.

For patients in the control group, the time from injury to surgery was prolonged until soft tissue swelling subsided. A tourniquet was helpful to diminish blood loss. Via a sinus tarsi approach, the reduction of the posterior facet could be easily achieved under direct visualization. The fracture fragments were fixed with a calcaneal plate and screws.

After the surgery, all patients received usual care. Partial weight-bearing exercises with crutches were permitted after 6 weeks and full weight-bearing activities after 12 weeks.

### Outcome assessment

The demographic variables, including gender, age, side of injury, Sanders classification, and type of injury, were collected. During each surgery, operation time and fluoroscopic times were recorded. At one, two, three, and six months of follow-up, radiological assessments were performed for all patients. Radiological imaging encompassed lateral and axial heel views, on which the calcaneal length, width, height, Bohler’s angle, and Gissane angle were measured. At six months of follow-up, functional outcomes were assessed with the use of the American Orthopaedic Foot and Ankle Society (AOFAS) Ankle-Hindfoot Score system (scores range from 0 to 100, with higher scores indicating better functioning). The occurrence of wound complications was recorded throughout the follow-up.

### Statistical analysis

We used the intention-to-treat principle to manage data missing. Two independent practitioners (HX and YanLi) conducted statistical analysis with the use of the SPSS software (version 26.0, Chicago, USA). Categorical data were calculated using the chi-square test or Fisher’s exact test. The Shapiro-Wilk method was used to test the normality of quantitative data. A two-sample t-test was used to compare normally distributed data between two groups, while the Mann-Whitney U test was applied for non-normally distributed data. P<0.05 was considered statistically significant.

## Results

A total of 32 patients were enrolled (19 in the 3D group versus 13 in the control group). There were no differences between groups in demographic variables. No crossovers occurred during the operation. The mean time from injury to surgery in the 3D group was three days less than that in the control group (*P* <0.001). Significant differences were detected between the 3D group and control group in operative duration (53.63±8.95 min, 95.08±8.31 min, *P* <0.001), fluoroscopic times (7.37±1.21, 16.85±1.57, *P* <0.001). At a follow-up of six months, the 3D group showed better restoration than the control group in calcaneal width, height, Bohler angle, and AOFAS Ankle-Hindfoot scores (all *P* <0.001). There were no significant between-group differences shown in calcaneal length and Gissane angle (*P* >0.05).

No wound-related complications occurred in all patients. There was no occurrence of iatrogenic vascular or nerve injury, fixation failure, or complication-related hardware removal. The comparative outcomes between groups are listed in Table 2.

**Table 2:** Comparative outcomes between two groups in the perioperative duration and at follow-up of six months

	3D group (n = 19)	Control group (n = 13)	Statistics	*P* value
Time to surgery (days)	3.26±0.87	7.23±2.09	*Z*=-4.179	<0.001
Intra-operative assessment				
Operative duration (min)	53.63±8.95	95.08±8.31	*t* = 13.553	<0.001
Fluoroscopic times	7.37±1.21	16.85±1.57	*Z*=-4.770	<0.001
Hospital stay (days)	9.00±1.70	14.77±1.36	*Z*=-4.757	<0.001
Postoperative radiography				
Calcaneal length (mm)	83.37±1.17	83.62±1.12	*t* = 0.598	0.554
Calcaneal width (mm)	34.00±1.76	38.15±1.46	*t* = 6.993	<0.001
Calcaneal height (mm)	47.26±1.59	42.00±2.20	*t* = 7.865	<0.001
Bohler angle (degree)	27.79±1.13	25.69±1.65	*t* = 4.267	<0.001
Gissane angle (degree)	128.63±3.10	126.62±2.33	*t* = 1.991	0.056
AOFAS score	79.90±2.40	74.31±4.21	*t* = 4.327	<0.001

## Discussion

The present study suggests that the 3D-printed cast assisted percutaneous screw fixation of DIACFs would yield superior outcomes in hospital stay, operative duration, fluoroscopic exposure, morphological restoration of the calcaneus, and functional recovery.

The screw configuration utilized in this study showed excellent biomechanical stability. In treating DIACFs, the primary goal is to return the calcaneal length, width, and height [3, 5]. To date, various kinds of screw configurations have been proposed to improve the fixation stability of DIACFs [13, 16–19]. For Sanders type II and III fractures, there is a consensus that the sustentaculum tali should be fixed with one or two screws. From an anatomical perspective, the sustentaculum tali, as a medial projection confluent with calcaneal tuberosity, composes the middle facet and bears subtalar stress. The high bone density of sustentaculum tali could ensure rigid purchase with screws [20, 21]. Therefore, the middle facet congruity and calcaneal width could be restored via the insertion of sustentaculum screws. In this study, a transverse screw was inserted 5 mm below the posterior facet, to the medial subcortical bone of sustentaculum tali. In this procedure, screws of a maximum of 3.5 mm are generally recommended. It is critical to avoid protruded tips into the subtalar joint space. In this study, the second group of screws was placed longitudinally to support the posterior facet. In previous studies, the posterior facet was supported by either oblique screws inserted from the inferior tuberosity, or parallel longitudinal screws [9, 11]. However, the height of the posterior facet was not effectively maintained by these methods. Loss of calcaneal height occurs frequently after surgery. Tomese et al. [2] proposed a crossed screw configuration and reported satisfactory outcomes. In this study, we made a minor modification to this crossed configuration. Specifically, two screws proceeded from the calcaneal tuberosity superiorly to the direction of the inferior calcaneocuboid joint, forming an X-shaped configuration. The insertion site was located much more superiorly, aiming to support the subtalar facet more effectively. In addition, the distance between insertion sites was adjusted to ensure the junction of screws just located beneath the midpoint of the posterior and middle facets. Thus, the crossed screws acted as rafting screws, which could be possible to provide optimal biomechanical stability. The third group of screws was placed parallelly in the longitudinal direction, aiming to correct calcaneal shortening and varus deformity. In the present study, the five screws were presented to be a three-dimensional configuration in the horizontal, coronal, and sagittal planes. Theoretically, this novel configuration could yield superior biomechanical stability over traditional plate fixation.

The 3D-printed cast showed another advantage in efficiency and accuracy during surgery. There were concerns that calcaneal fragments would migrate in the reduction procedure, which may result in a mis-insertion with the presigned jig. In the present study, we chose the sustentaculum tali and lateral malleolus as the reference to design 3D cast, the former of which is regarded as the “constant” fragment in DIACFs. Gitajn et al. [17] analyzed 212 CT images of patients with DIACFs and revealed that sustentacular fractures were detected in 44.3% of DIACFs. Moreover, 76.6% of sustentacular fractures showed no displacement. The sustentaculum tali and talus were anatomically aligned in 78.3% of DIACFs. Given that the sustentaculum tali stay constantly in a high proportion in DIACFs, the sustentaculum tali is reliable to guide screw insertion. In this study, all screws were inserted successfully as planned and no intra-operative injury of the medial planter nerve occurred. We took several measures to improve the compatibility of the 3D-printed jig. First, DIACFs with displaced or comminuted sustentaculum tali were excluded from the study. Second, the CT scans and cast printing were completed within two days before surgery, aiming to minimize the potential impact of subsided soft tissue swelling on the jig use. Third, the coverage of the 3D cast was expanded to the lateral malleolus and distal plantar, which could improve the stability of the 3D printed cast on foot.

The screw placement was efficient with the use of a 3D-printed cast. The volume of the calcaneus is relatively small. In traditional surgery, as several prior screws have been placed, additional screws are difficultly inserted. Multiple adjustments are frequently carried out to achieve satisfactory placement, which increases operative time and fluoroscopic exposure. This problem could be resolved with the assistance of the 3D-printed cast. In this study, the 3D group showed much shorter operative time and less fluoroscopic times than the control group.

The current research has several limitations. First, the sample size is small. Second, the selection of fixation methods was based on the discretion of the surgeon, which may generate selection bias. Third, the follow-up time is short and may compromise the conclusions. Fourth, the additional cost of the 3D-printed cast may restrict its clinical application.

## Conclusions

In conclusion, according to the preliminary comparison of 3D-printed cast assisted screw fixation and minimally invasive plate fixation for DIACFs, the 3D-printed cast had shown superiority in operative duration, fluoroscopic exposure, morphological restoration, and functional outcomes. The indication for 3D-printed assisted screw fixation is Sanders type II, III DIACFs. The fabrication of the 3D-printed cast within two days before surgery can contribute to improving the compatibility of the calcaneal jig. It is a safe and effective approach for the treatment of Sanders type II and III calcaneal fractures and is worthy of clinical promotion.

## Data Availability

The datasets used and/or analyzed during the current study are available from the corresponding author on reasonable request.

## Abbreviations

DIACFs: Displaced intra-articular calcaneal fractures
3D: Three-dimensional
AOFAS: American Orthopaedic Foot and Ankle Society
CT: Computed tomography
