# Correction: Three-dimensional printed cast assisted screw fixation of calcaneal fractures: a prospective study

**DOI:** 10.1186/s12891-023-07118-x

**Published:** 2023-12-30

**Authors:** Qizhi Song, Tao Li, Huan Xia, Yan Li, Chengbin Feng, Yajun Lin, Huahong Wang, Jinbiao Hu, Qilong Jiang

**Affiliations:** 1 Department of Orthopaedic Surgery, Chonggang General Hospital, Chongqing, China; 2 Nursing Department, Chonggang General Hospital, Chongqing, China; 3 Central Sterile Supply Department, Chonggang General Hospital, Chongqing, China; 4 Department of Orthopaedic Surgery, Chongqing Orthopedic Hospital of Traditional Chinese Medicine, No. 9, Jiefang West Road, Chongqing, 400010 China

**Correction:** ***BMC Musculoskelet Disord*** **24, 802 (2023)**

10.1186/s12891-023-06927-4

Following publication of the original article [1], the asterisk symbol appeared from Qizhi Song’s name in the author group which indicates corresponding author was removed. Qilong Jiang is the only corresponding of this article.

The original article [1] has been updated.
